# Reclassification of eight *Akkermansia* *muciniphila* strains and description of *Akkermansia* *massiliensis* sp. nov. and *Candidatus* Akkermansia timonensis, isolated from human feces

**DOI:** 10.1038/s41598-022-25873-0

**Published:** 2022-12-16

**Authors:** Sokhna Ndongo, Nicholas Armstrong, Didier Raoult, Pierre-Edouard Fournier

**Affiliations:** 1 MEPHI, IHU Méditerranée Infection, Aix Marseille University, 19-21 Boulevard Jean Moulin, 13005 Marseille, France; 2 IHU-Méditerranée Infection, Marseille, France; 3 IRD, AP-HM, SSA, VITROME, IHU-Méditerranée Infection, Aix Marseille University, Marseille, France; 4 Special Infectious Agents Unit, King Fahd Medical Research Center, King Abdulaziz University, Jeddah, Saudi Arabia

**Keywords:** Microbiology, Bacterial evolution, Bacterial genomics

## Abstract

*Akkermansia* *muciniphila* is a human intestinal tract bacterium that plays an important role in the mucus layer renewal. Several studies have demonstrated that it is a modulator for gut homeostasis and a probiotic for human health. The *Akkermansia* genus contains two species with standing in nomenclature but their genomic diversity remains unclear. In this study, eight new *Akkermansia* sp. strains were isolated from the human gut. Using the digital DNA-DNA hybridization (dDDH), average nucleotide identity (ANI) and core genome-based phylogenetic analysis applied to 104 *A.* *muciniphila* whole genomes sequences, strains were reclassified into three clusters. Cluster I groups *A.* *muciniphila* strains (including strain ATCC BAA-835^T^ as type strain), whereas clusters II and III represent two new species. A member of cluster II, strain Marseille-P6666 differed from *A.* *muciniphila* strain ATCC BAA-835^T^ and from *A.* *glycaniphila* strain Pyt^T^ in its ability to grow in microaerophilic atmosphere up to 42 °C, to assimilate various carbon sources and to produce acids from a several compounds. The major fatty acids of strain Marseille-P6666 were 12-methyl-tetradecanoic and pentadecanoic acids. The DNA G + C content of strain Marseille-P6666 was 57.8%. On the basis of these properties, we propose the name *A.* *massiliensis* sp. nov*.* for members of cluster II, with strain Marseille-P6666^T^ (= CSUR P6666 = CECT 30548) as type strain. We also propose the name “*Candidatus* Akkermansia timonensis” sp. nov. for the members of cluster III, which contains only uncultivated strains, strain Akk0196 being the type strain.

## Introduction

The human gastro-intestinal tract is a complex ecosystem in which lives a dynamic microbial community. Most of this microbiota is represented by bacteria that play a crucial role in the body physiological balance, with an important impact on immunity, various metabolic processes and host health^1^. *Akkermansia* *muciniphila*, described in 2004 by Derien et al.^2^, is a bacterium able to use mucin as sole carbon source. It participates in the renewal of mucus and the integrity of the intestinal barrier^3^. It is a commensal of the intestinal flora (3–5%)^4^ and its abundance was suggested as a biomarker of an healthy gut microbiota^5,6^. In contrast, the number of studies depicting its decrease or depletion in inflammatory bowel diseases, such as Crohn's disease, colorectal cancer, ulcerative colitis^7^ and metabolic diseases including obesity^8^ and type 2 diabetes^9^, is constantly increasing. The reconstitution of its abundance by oral administration (murine model) has been correlated to several beneficial effects such as a reduction of hepatic injury, steatosis and neutrophil infiltration in animals with alcoholic liver disease^10^, to the decrease of atherosclerotic lesions and the metabolic endotoxemia-induced inflammation^11^, to an attenuation of dextran sulfate sodium (DSS)-induced acute colitis^12^, to the suppression of colonic tumorigenesis in ApcMin/+ mice, and to a reduced growth of implanted HCT116 or CT26 tumors in nude mice^13^.

*Akkermansia* *muciniphila* also possesses beneficial probiotic activities on the mucus layer^3,14^. Supplementation in *A.* *muciniphila* was demonstrated to significantly reduce the severity of nausea, vomiting, constipation during pregnancy, to play a key role in the regulation of metabolic functions to prevent obesity^15,16^, and to enhance glucose tolerance and attenuate adipose tissue inflammation^17^ and reduce diabetes incidence^18^.

The *Akkermansia* genus contains two validated species, i.e., *A.* *muciniphila* and *A.* *glycaniphila*^2,19^. In 2021, two putative new *Akkermansia* species*,* “*Candidatus* A. intestinavium” and “*Candidatus* A. intestinigallinarum” were described by Gilroy et al*.*^20^. Kim et al*.* observed a great genomic diversity within the species *A.* *muciniphila*^21^. Likewise, Orellana et al., described a potentially new genus within the family *Akkermansiaceae*, “*Candidatus* Mariakkermansia forsetii”, from a metagenomic analysis of surface seawater samples^22^. However, despite its role as a probiotic confirmed by numerous studies and its benefits on host health, very few *Akkermansia* species are described. In a project to search for new *Akkermansia* *muciniphila* strains and analyze their genomic diversity, we discovered two strains that may be members of a new *Akkermansia* species. Here, we report a taxonogenomic description of this new species and its phenotypic and biochemical characteristics. An analysis of complete *A.* *muciniphila* genomes available in public databases as well as those from eight isolates from our collection, allowed us to reclassify strains within this genus.

## Results and discussion

### Strain identification and phylogenetic analyses

All strains isolated in this study were first identified as *Akkermansia* *muciniphila* by MALDI-TOF–MS when we comparing their peptidic profiles to those available in the Bruker database. After sequencing the 8 strains, the 16S rRNA sequences of each isolate were extracted and compared to those of closely related species in the NCBI database (https://www.ncbi.nlm.nih.gov/). Strains Marseille-P6666, Marseille-P9185, Marseille-P5162, Marseille-P6566, Marseille-P7245, Marseille-P9184, Marseille-P9642 and Marseille-Q2586 showed highest 16S rRNA sequence similarities of 99.20, 98.95, 99.73, 99.79, 99.72, 99.96, 99.79 and 99.86%, respectively, with *A.* *muciniphila* strain ATCC BAA-835^T^ (AY271254).

### Phenotypic and biochemical analysis

**Figure 1:**
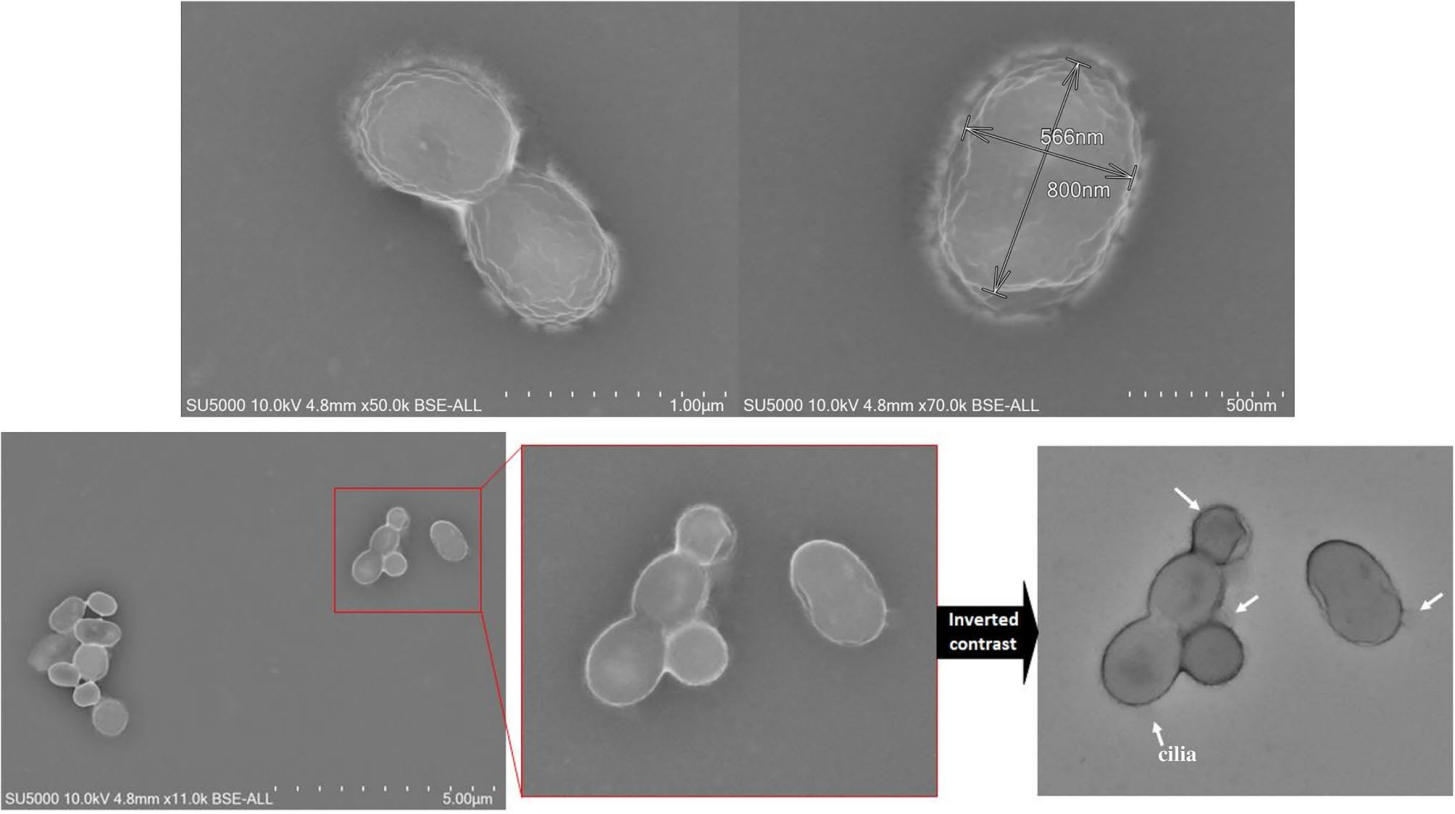
Transmission electron microscopy of *Akkermansia* *massiliensis* strain Marseille-P6666.

Cells from strain Marseille-P6666 were rod-shaped (0.5 × 0.8 μm), motile and Gram-negative (Fig. 1). In the presence of fluid, the cells turn on themselves and self-propel. Multiple cilia can be observed on the bacterial cell surface (Fig. 1). Colonies grown on Columbia agar plates appeared white, non-haemolytic, and circular with a diameter of 0.5 mm after 72 h of incubation. Optimal growth from strain Marseille-P6666 grew between from 37 to 42 °C, at a pH ranging from 6 to 7.5, and in the presence of 0 to 5 g/l NaCl. Strain Marseille-P6666 was able to grow in microaerophilic atmosphere, which enables it to survive in the mucus layer of the gastrointestinal tract^23^. The new isolate was able to use mucin as a solo carbon source.

Cells were catalase-positive and oxidase-negative. Using an API ZYM strip (bioMérieux), production of alkaline phosphatase, esterase (C4), acid phosphatase, naphthol-AS-BI-phosphohydrolase, α-galactosidase, β-galactosidase, β-glucuronidase and *N*-acetyl-β-glucosaminidase were positive. Using an API 20NE strips (bioMérieux), strain Marseille-P6666 was able to hydrolyze esculin and to produce β-galactosidase. Using an API 20A strip (bioMérieux), positive reactions were obtained for acidification of d-glucose, d-mannitol, d-lactose, d-maltose, esculin ferric citrate and d-mannose. Using an API 50CH strips (bioMérieux), strain Marseille-P6666 utilized l-arabinose, d-ribose, d-galactose, d-glucose, d-fructose, d-mannose, d-mannitol, d-sorbitol, *N*-acetylglucosamine, salicin, esculin ferric citrate, cellobiose, d-maltose, d-lactose, d-melibiose, amygdaline, d-saccharose, d-trehalose, arbutine, gentiobiose, d-turanose, d-tagalose and potassium gluconate as sole carbon sources. All negative properties obtained from the API ZYM, 50CH, 20A and 20NE strips were summarized in the description of the novel species. Furthermore, the physiological and biochemical characteristics of strain Marseille-P6666^T^ were summarized and compared to those of other closely related species in Table 1. Strain Marseille-P6666 was found to be susceptible to trimethoprim-sulfamethoxazole, doxycycline, rifampicin, clindamycin, amoxicillin, oxacillin and benzylpenicillin but susceptible to vancomycin, amikacin, ciprofloxacin, tobramycin, ceftriaxone and ceftazidime. The major cellular fatty acids of strain Marseille-P6666 were saturated structures: 12-methyl-tetradecanoic acid (58.4%), pentadecanoic acid (15.8%) and 12-methyl-Tridecanoic acid (6%). The two major fatty acid namely anteiso-C_15:0_ and C_15:0_ are similar for strains Marseille-P6666, Muc^T^ and Pyt^T^ (Table 2). Short fatty acids such as acetic acid (12 ± 7 mM) and propanoic acid (5 ± 2 mM) were produced. Strain Marseille-P6666 produced various polar lipids classes such as sphingomyelins, N-acyl ethanolamines, acyl carnitine, phosphatidylethanolamine, Lysophosphatidyléthanolamine, phosphatidylcholine, lysophosphatidylcholine, ceramides—glycero lipids, fatty acyls—glycero lipids, phosphatidic acid and several unknown structures and phospholipids (Supplementary Fig. 1 and Supplementary Table 1).

**Table 1:** Physiological features of *Akkermansia* *massiliensis* strain Marseille-P6666 (a), *Akkermansia* *muciniphila* strain ATCC BAA-835^T^ (b), *Akkermansia* *glycaniphila* strain Pyt^T^ (c), *Verrucomicrobium* *spinosum* strain DSM4136^T^ (d), *Prosthecobacter* *debontii* strain ATCC 700200^T^ (e); (+): positive; (−): negative; (*): weak reaction; *NA* not available data.

Properties	a	B	C	d	E
Size (μm)	0.5 × 0.8 μm	0.6 × 1.0	0.6 × 1.0	0.8–1.0 × 1.0–3.8	2–8 × 0.5
Gram	−	−	−	−	−
Tolerance to oxygen	+	−	−	+	−
Motility	+	−	−	−	−
Endospore formation	−	−	−	NA	NA
Catalase	+	NA	+	+	NA
Nitrate reductase	−	NA	NA	−	NA
Urease	−	NA	−	+	NA
Indole production	−	NA	−	NA	NA
Aesculin hydrolysis	+	NA	+	+	NA
β-galactosidase	+	NA	NA	NA	NA
*N*-acetylglucosamine	+	+	+ *	+	NA
Ribose	+	NA	NA	−	+
Arabinose	−	NA	−	−	−
Rhamnose	−	−	−	+	+
Raffinose	−	NA	−	+	−
Inulin	−	NA	NA	−	NA
Maltose	+	−	+	+	+
Cellobiose	+	−	−	+	+
Melezitose	−	NA	−	+	NA
Melibiose	−	NA		+	+
Trehalose	+	NA	−	+	+
Galactose	+	−	+ *	+	+
d-glucose	+	+	+ *	+	+
d-fructose	+	−	−	+	−
Mannose	+	NA	−	+	+
Mannitol	+	NA	−	−	NA
Xylose	−	−	+	+	+
Lactose	+	−	+	+	+
Major faty acid product	Anteiso-C_15 : 0_	Anteiso-C_15 : 0_	Anteiso-C_15 : 0_	NA	NA
DNA G + C content (mol%)	57.8	55.6	58.2	57.9–59.3	57.1

**Table 2:** Cellular fatty acid composition (%) of *Akkermansia* *massiliensis* strain Marseille-P6666, *Akkermansia* *muciniphila* strain ATCC BAA-835^T^ and *Akkermansia* *glycaniphila* strain Pyt^T^.

Fatty acids	Name	Mean relative %^a^		
		1	2	3
C 15:0 anteiso	12-Methyl-tetradecanoic acid	58.4 ± 6.1	53.6	42.3
C 15:0	Pentadecanoic acid	15.8 ± 3.4	9.2	13.1
C 14:0 iso	12-Methyl-tridecanoic acid	6.0 ± 1.5	3.1	1.3
C 16:0	Hexadecanoic acid	6.0 ± 0.5	4	10.7
C 17:0	Heptadecanoic acid	5.2 ± 1.2	6.3	0.5
C 18:0	Octadecanoic acid	1.8 ± 0.2	1.1	1.1
C 16:0 iso	14-Methyl-pentadecanoic acid	1.8 ± 0.2	0.5	0.6
C 17:0 anteiso	14-Methyl-hexadecanoic acid	1.5 ± 0.2	4.5	2.5
C 15:0 iso	13-Methyl-tetradecanoic acid	TR	2.6	1.2
C 18:1n9	9-Octadecenoic acid	TR	-	-
C 16:1 iso	14-Methyl-pentadecenoic acid	TR	-	-
C 14:0	Tetradecanoic acid	TR	0.7	3.9
C 13:0	Tridecanoic acid	TR	0.2	0.2
C 18:2n6	9,12-Octadecadienoic acid	TR	−	−
C 18:1n7	11-Octadecenoic acid	TR	−	−
C 13:0 anteiso	10-Methyl-dodecanoic acid	TR	−	−
C 12:0	Dodecanoic acid	TR	−	−

### Genome analysis and comparison

The genome properties of the eight strains sequenced in this study are summarized in Table 3. Genome sequences of these isolates had different sizes ranging from 2,740,501 to 3,280,190 bp.

**Table 3:** Genomic characteristics of studied *Akkermansia* species and other closely-related species.

Strain name	Genbank accession	Size, Mb	G + C mol%	Gene count
Marseille-P6666	JAMGSI000000000	3,280,190	57.8%	2,793
Marseille-P9185	JAMYIA000000000	3,193,520	57.9%	2,672
Marseille-P5162	JAMZOC000000000	2,997,637	55.1%	2,648
Marseille-P6566	JAMZOB000000000	2,784,480	55.2%	2,402
Marseille-P7245	JAMGSH000000000	2,784,249	55.4%	2,381
Marseille-P9184	JAMGSG000000000	2,814,301	55.6%	2,471
Marseille-P9642	JAMGSF000000000	2,740,501	55.7%	2,343
Marseille-Q2586	JAMGSE000000000	2,812,384	55.8%	2,422
ATCC BAA-835^T^	CP001071.1	2,664,051	55.6%	2,183
Pyt^T^	LIGX00000000	3,074,078	58.2%	2,527
ChiGjej6B6-8097	DXEH00000000.1	2,313,406	65.09%	1,836
14975	DXFQ00000000.1	2,129,131	63.4%	1,813
Akk0196	CP072051.1	3,212,887	56.7%	2,705
Akk7	20100303_Bin_52_1	2,025,900	51.1%	1,838

**Figure 2:**
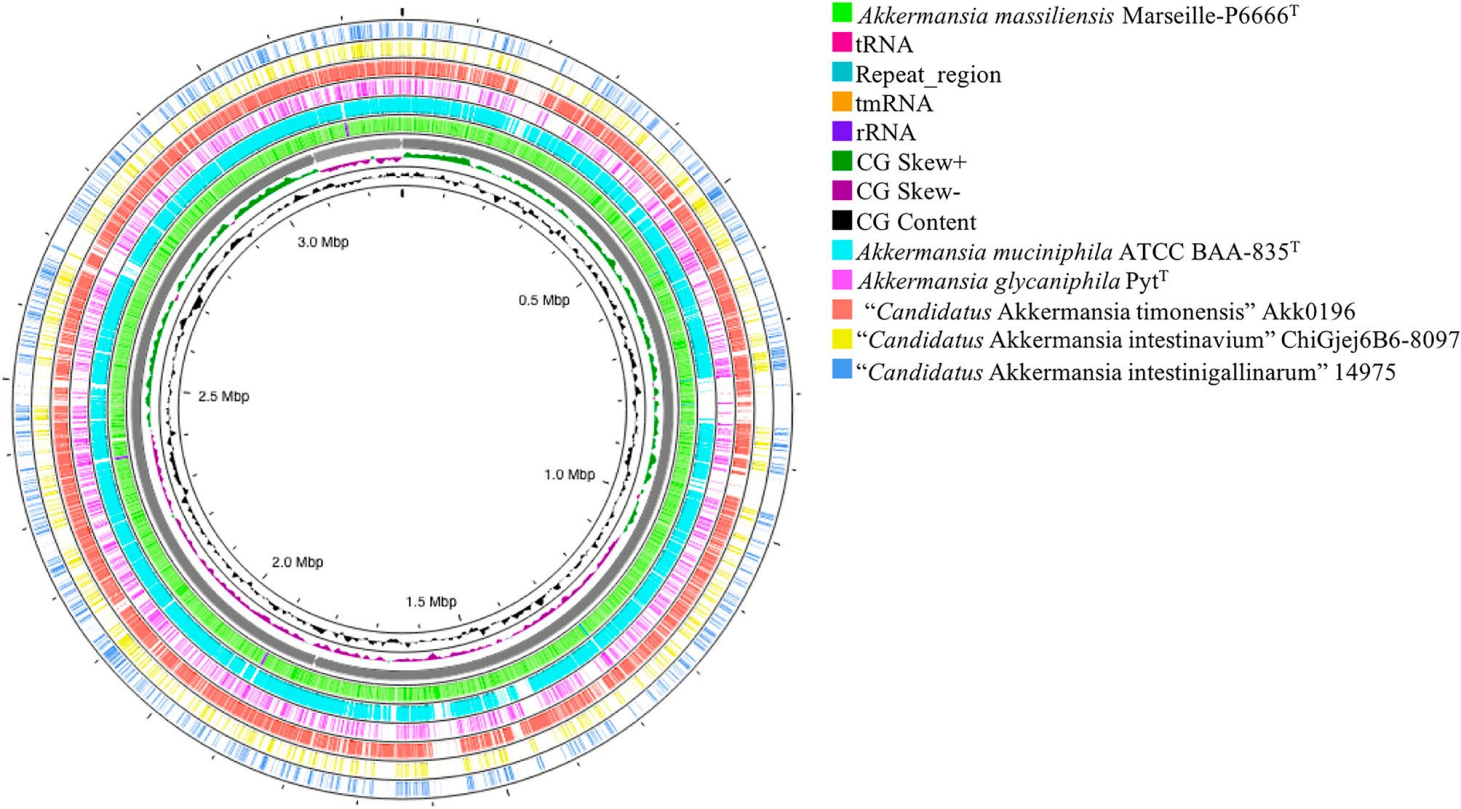
Graphical circular map of *A.* *massiliensis* Marseille-P6666 uses as reference genome to compare the genomic organization of the closest species of published *Akkermansia* such as *Akkermansia* *muciniphila* strain ATCC BAA-835^T^, *Akkermansia* *glycaniphila* strain Pyt^T^, *Candidatus* Akkermansia intestinavium” strain ChiGjej6B6-8097^T^ and *Candidatus* Akkermansia intestinigallinarum” strain 14975^T^, and the new species candidate “*Candidatus* Akkermansia timonensis” strain Akk0196. The different colors used in the circular map are indicated in the legend on the right part of the figure.

**Figure 3:**
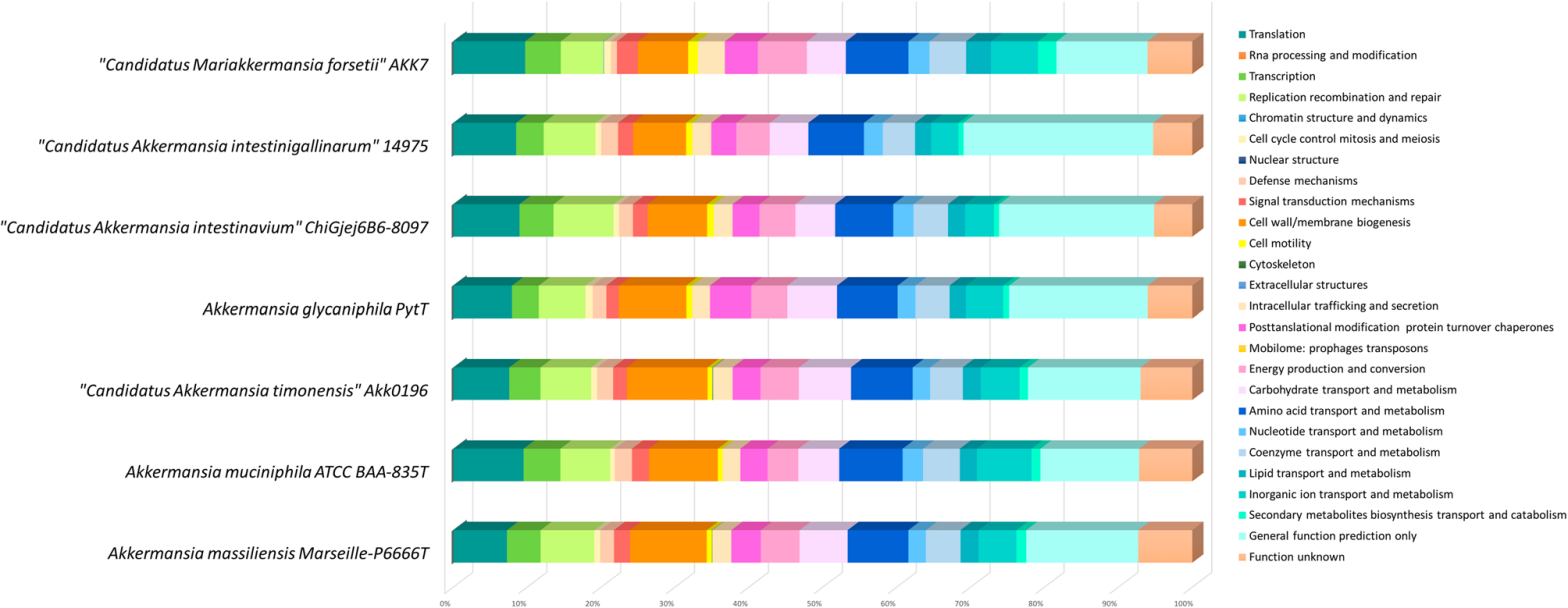
Distribution of predicted protein-coding genes from *Akkermansia* *muciniphila* strain ATCC BAA-835^T^ (Cluster I), *Akkermansia* *massiliensis* strain Marseille-P6666 (Cluster II), “*Candidatus* Akkermansia timonensis” strain Akk0196 (Cluster III), *Akkermansia* *glycaniphila* strain Pyt^T^, and other closely-related species, in COG categories.

The genome of Marseille-P6666 is 3,280,190 bp long with an average G + C content of 57.8%. It is composed of three contigs. Of the 2793 predicted genes, 2726 were protein-coding genes, 9 RNAs, 1 tmRNA and 57 tRNA genes. Circular maps of this strain are illustrated in Fig. 2. The genome of Marseille-P6666 is greater than that of *A.* *muciniphila* ATCC BAA-835^T^ (= Muc^T^), (2,664,051 bp) and *A.* *glycaniphila* pyt^T^ (3,074,078 bp). The G + C content of Marseille-P6666 is larger than that of *A.* *muciniphila* ATCC BAA-835^T^ (55.6%) but smaller than that of *A.* *glycaniphila* Pyt^T^ (58.2%). Distribution of genes into COGs functional categories between Marseille-P6666 and the other closely related species was presented in Fig. 3 and Table 4. The number of genes from each COG category was greater for strain Marseille-P6666 than for *A.* *muciniphila* strain ATCC BAA-835^T^, notably for genes encoding cell wall and membrane biogenesis, energy production and conversion, defense mechanisms, and transport and metabolism of carbohydrates, amino acids, nucleotides and inorganic ions. This is consistent with the fact that strain Marseille-P6666 has more coding genes (1983 genes) than *A.* *muciniphila* strain ATCC BAA-835^T^ (1084 genes). The phylogenetic tree based on core genome identified tree clusters (I, II, and III), with strains considered previously belonging to the *A.* *muciniphila* species. Among the *Akkermansia* strains isolated in this study, six of them, clustered with *A.* *muciniphila* strain ATCC BAA-835^T^ (Cluster I). Two strains formed a second cluster (Cluster II) with other strains previously classified as belonging to the *A.* *muciniphila* species. Comparison of the genomes from members of the clusters I, II and III with *A.* *muciniphila* strain ATCC BAA-835^T^, showed dDDH values higher than 70% with cluster I members (range 74.80% to 100%) but lower than 70% for cluster II and III members (range 33.8–34.2% and 17.1–24.9%, respectively, Supplementary Table 2). ANI values between cluster I, II and III isolates were 97–100%, 88% and 82% with *A.* *muciniphila* ATCC BAA-835^T^, respectively (Supplementary Fig. 2). ANI values between the three clusters (I, II and III) were significantly lower than the proposed cutoff value of 95% for defining a bacterial species^24,25^. A recent study even redefined the ANI threshold value to 96.5% for creating a new bacterial species^26^. In contrast, all ANI values within a given cluster were higher than 97%. Therefore, the distribution of strains previously considered as *A.* *muciniphila* into three distinct species was clearly supported by the genome-based phylogenetic analysis and the of DDH and ANI values.

**Table 4:** Numbers of genes associated with the 25 functional categories in clusters of orthologous groups (COG).

Description	COG	1	2	3	4	5	6	7
Translation	[J]	144	103	147	151	140	130	133
Rna processing and modification	[A]	0	0	0	0	0	0	0
Transcription	[K]	90	54	82	69	72	57	66
Replication, recombination and repair	[L]	144	73	133	120	127	107	79
Chromatin structure and dynamics	[B]	0	0	0	0	0	0	1
Cell cycle control, mitosis and meiosis	[D]	16	7	16	19	12	12	13
Nuclear structure	[Y]	0	0	0	0	0	0	0
Defense mechanisms	[V]	37	25	42	35	29	35	11
Signal transduction mechanisms	[T]	43	25	36	32	31	31	39
Cell wall/membrane biogenesis	[M]	206	101	212	174	126	110	93
Cell motility	[N]	14	7	13	15	14	13	18
Cytoskeleton	[Z]	1	0	1	0	0	0	0
Extracellular structures	[W]	0	0	1	0	0	0	0
Intracellular trafficking and secretion	[U]	51	26	51	46	40	39	50
Posttanslational modification, protein turnover,chaperones	[O]	79	40	73	106	57	52	61
Mobilome: prophages, transposons	[X]	0	0	0	0	0	0	0
Energy production and conversion	[C]	104	45	101	93	76	69	91
Carbohydrate transport and metabolism	[G]	129	60	137	128	84	80	72
Amino acid transport and metabolism	[E]	163	93	162	156	123	115	116
Nucleotide transport and metabolism	[F]	47	30	46	46	43	39	39
Coenzyme transport and metabolism	[H]	93	54	86	88	73	67	68
Lipid transport and metabolism	[I]	48	25	47	42	36	33	46
Inorganic ion transport and metabolism	[P]	102	80	102	96	61	57	87
Secondary metabolites biosynthesis, transport and catabolism	[Q]	26	13	22	15	11	10	34
General function prediction only	[R]	301	145	296	357	328	393	169
Function unknown	[S]	145	78	136	115	81	81	83

*Akkermansia* *massiliensis* strain Marseille-P6666 (1), *Akkermansia* *muciniphila* strain ATCC BAA-835^T^ (2), *Candidatus* Akkermansia timonensis strain Akk0196 (3), *Akkermansia* *glycaniphila* strain Pyt^T^ (4), “*Candidatus* Akkermansia intestinavium” strain ChiGjej6B6-8097^T^ (5), “*Candidatus* Akkermansia intestinigallinarum” strain 14975^T^ (6) and “*Candidatus* Mariakkermansia forsetii” strain AKK7 (7).

**Figure 4:**
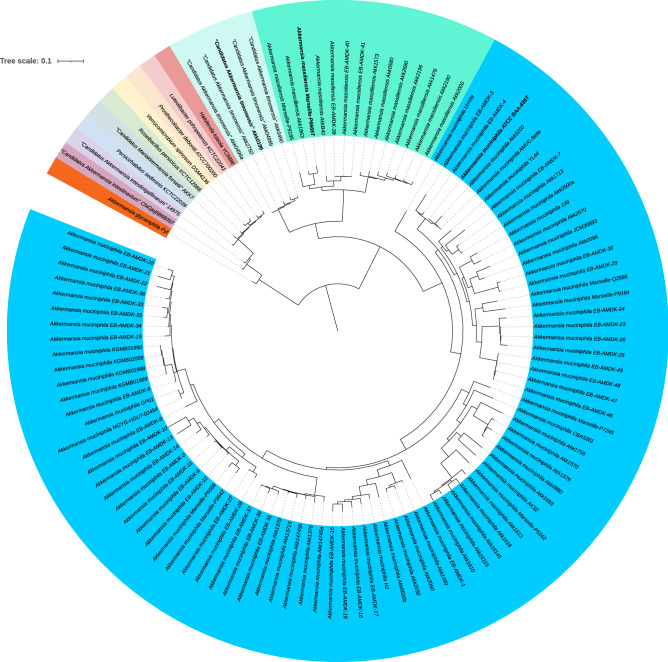
Core genome-based phylogenetic tree highlighting the position of *Akkermansia* *muciniphila* strain ATCC BAA-835^T^ (Cluster I), *Akkermansia* *massiliensis* strain Marseille-P6666 (Cluster II), “*Candidatus* Akkermansia timonensis” strain Akk0196 (Cluster III), relative to other type strains within the genus *Akkermansia* and other members of the family *Verrucomicrobiaceae*. Sequence alignment was performed using MUGSY and a phylogenetic tree was obtained using the maximum-likelihood method. Numbers at the nodes are bootstrap values (≥ 70%) obtained by repeating the analysis 1,000 times. The scale bar indicates a 10% nucleotide sequence divergence.

Hence, our genome analysis results strongly suggest that strains currently classified as *A.* *muciniphila* belong to three distinct species. However, the 98.7% 16SrRNA sequence similarity threshold defined to classify a bacterial species^27^ cannot discriminate between species in the *Akkermansia* genus. This highlights the limitation of 16S rRNA gene analysis for the correct species classification within some bacterial genera (Supplementary Fig. 3)^28^. Cluster I is formed by *A.* *muciniphila* strains, including the type strain ATCC BAA-835^T^ (Fig. 4)*.* Cluster II includes strains Marseille-P6666, Marseille-P9185 and 12 other strains described in previous studies (Fig. 4). Recently, Kumar et al.^29^ described a new *Akkermansia* strain, DSM 33459, that was phylogenetically close to strains EB-AMDK-39, EB-AMDK-40, and EB-AMDK-41, and proposed that this strain belongs to a new *Akkermansia* species. According to these authors, genomic comparison showed that strain *Akkermansia* *sp*. DSM 33459) exhibited OrthoANI values > 98% with strains EB-AMDK-39, EB-AMDK-40, and EB-AMDK-41, thus classifying it within the same species. This result also indicates that *Akkermansia* sp. strain DSM 33459 belongs to the same cluster, and probably to the same species, as strains Marseille-P6666 and Marseille-P9185. However, no accession number was provided by Kumar et al. for the genomic sequence of *Akkermansia* sp. strain DSM 33459, and therefore could not be included in our analysis. In addition, the authors deposited strain DSM 33459 in the DSMZ collection, but not in a second culture collection as requested by rule 30 from the international code of nomenclature of Prokaryotes for the description of a new species^30^. In addition, no name was proposed by the authors for this new species and Kumar and colleagues’ article does not contain any protolog to officially describe the properties of the new species. Cluster III includes the five strains Akk0196^T^, Akk0490, Akk0496a, Akk0496b and Akk2750 (Fig. 4).

We observed that *A.* *glycaniphila* strain Pyt^T^, initially described by Janneke et al., exhibited dDDH and ANI values of 22.5% and 73%, respectively, with *A.* *muciniphila* strain ATCC BAA-835^T^^19^. In addition, *A.* *glycaniphila* strain Pyt^T^ also exhibited dDDH and ANI values ranging from 18.5 to 24% and from 73 to 74%, respectively, with all other members of the *Akkermansia* genus.

### Pan-genome analysis of *Akkermansia muciniphila, Akkermansia massiliensis* sp. nov. and *Candidatus* Akkermansia timonensis sp. nov*.*

The pan- and core-genomes of *A.* *muciniphila* strains (85 strains) were composed of 6357 and 1193 genes, respectively. In addition, 1654 genes are accessory genes. A total of 1108 specific genes were found in only one *A.* *muciniphila* strain.

The pan- and core-genomes of *A.* *massiliensis* strains (14 strains) were composed of 3632 and 2138 genes, respectively. The accessory genome included 410 genes were strain-specific.

The pan- and core-genomes of *A.* *timonensis* strains (5 strains) were composed of 2767 and 2539 genes, respectively. The accessory genome sizes were 228 genes. A total of 125 specific genes were found in only one strain of *A.* *timonensis.*

The pan-genome of *A.* *muciniphila* ATCC BAA-835^T^ (6357genes) is larger than that of *A.* *massiliensis* Marseille-P6666 (3632 genes). The percentage of the core-genome of *A.* *muciniphila* ATCC BAA-835^T^ is smaller than that of *A.* *massiliensis* Marseille-P6666, 18.7% and 60.1%, respectively. However, due to the difference in the number of genomes used in the analysis of each pan-genome, the number of accessory genes of *A.* *muciniphila* ATCC BAA-835^T^ (85 strains) is higher than that of *A.* *massiliensis* Marseille-P6666 (14 strains), 26% and 11.2% of the pan-genome, respectively.

## Conclusion

From these results, we suggested the creation of two new species: *Akkermansia* *massiliensis* sp. nov. that includes strains Marseille-P6666 and Marseille-P9185, for which Marseille-P6666^T^ is the type strain; and *Candidatus* Akkermansia timonensis sp. nov*.* that includes strains Akk0196, Akk0490, Akk0496a, Akk0496b and Akk2633.

### Description of *Akkermansia massiliensis* sp. nov.

*Akkermansia* *massiliensis* (mas.si.li.en'sis L. fem. adj. *massiliensis,* of Massilia, the Latin name of Marseille where the strain was isolated).

Gram strain-negative, rod-shaped cells (0.5 × 0.8 μm). Bacteria are catalase-positive, oxidase-negative and motile. Non-spore forming. Colonies grown on Columbia agar are white, circular, convex and with entire margins and uniform. The optimal growth is observed in anaerobic atmosphere, at 37 °C, at pH 7 and in the presence of 5 g/l NaCl. Growth may also be obtained in microaerophilc atmosphere and at temperatures up to 42 °C. Nitrate reduction, indole production, gelatin hydrolysis and urease activities are absent. Strain Marseille-P6666^T^ is positive for esculin hydrolysis and exhibits α-galactosidase, β-galactosidase, β-glucuronidase, *N*-acetyl-β-glucosaminidase, β-galactosidase, alkaline phosphatase, esterase (C4) Naphtol phosphohydrolase and acid phosphatase activities. Esterase lipase (C8), lipase (C14), leucine arylamidase, valine arylamidase, cystine arylamidase, trypsin α-chymotrypsin, α-glucosidase, β-glucosidase, α-mannosidase and α-fucosidase activities are not detected. The following substrates are used for growth and acid production: l-arabinose, d-ribose, d-galactose, d-glucose, d-fructose, d-mannose, d-mannitol, d-sorbitol, mucin, *N*-acetylglucosamine, salicin, esculin ferric citrate, cellobiose, d-maltose, d-lactose, d-melibiose, amygdaline, d-saccharose, d-trehalose, arbutine, gentiobiose, d-turanose, d-tagalose and potassium gluconate. Strain Marseille-P6666^T^ does not utilize glycerol, erythritol, d-arabinose, d-xylose, l-xylose, d-adonitol, methyl-α d-mannopyranoside Methyl-α d-glucopyranoside, l-sorbose, l-rhamnose, dulcitol, inositol, Methyl-α d-Glucopyranoside, Methyl-α d-Mannopyranoside, inulin, d-melezitose, d-raffinose, glycogen, amidon, d-fucose, l-fucose, d-arabitol, l-arabitol, potassium 2-cetogluconate and potassium 5-cetogluconate. The major fatty acids are 12-methyl-tetradecanoic acid, pentadecanoic acid. Acetic acid and propanoic acid are produced. Various polar lipids classes are found: sphingomyelins, *N*-acyl ethanolamines, acyl carnitine, phosphatidylethanolamine, lysophosphatidyléthanolamine, phosphatidylcholine, lysophosphatidylcholine, ceramides—glycero lipids, fatty acyls—glycero lipids, phosphatidic acid and several unknown structures and phospholipids.

The DNA G + C content of the genomic DNA is 57.8%. The type strain Marseille-P6666^T^ (= CSUR P6666 = CECT 30548), was isolated from human stool.

### Description of *Candidatus* Akkermansia timonensis sp. nov.

*Candidatus* Akkermansia timonensis (ti.mo.nen’sis. L. fem. adj. timonensis, of Timone, the name of the hospital where this genome was analyzed).

Member of the *Akkermansia* genus, *Candidatus* Akkermansia timonensis is a bacterial species identified by metagenomic analyses. The genome length of the type genome is 3,212,887 bp and the G + C content is 56.7%.

The type material is Akk0196, a metagenome-assembled genome from human stool.

## Materials and methods

### Isolation and identification of strains by MALDI-TOF

Stools obtained from eight French patients as part of a culturomics project aiming at isolating as many distinct human-associated bacterial species from the gut, were included in the study from 2017 to 2020. All the methods used in this study were carried out in accordance with relevant guidelines and regulations conformed to the Declaration of Helsinki. Informed and oral consent was obtained from the stool donors. Approximately 1 g of each feces specimen was suspended in 2 ml of phosphate-buffered-saline (Life, Technologies, Carisbad, CA, USA). Then, 100 µl of each stool suspension was tenfold diluted up to 10^–10^. After that, 50 µl was inoculated on 5% sheep blood-enriched Columbia agar (BioMérieux, Marcy l’Etoile, France) and incubated at 37 °C in anaerobic atmosphere generated by AnaeroGen generator (bioMérieux). After 72 h of incubation, single colonies were selected and subcultured on the same medium in order to obtain pure isolates. Strains were identified using a Microflex MALDI-TOF MS spectrometer (Bruker, Daltonics, Leipzig, Germany) as previously described^31^.

### Phenotypic and biochemical characterization

Cell morphology and characteristics of these isolates were observed using a TM4000 scanning electron microscope (Hitachi, Tokyo, Japan) from fresh colonies as previously described^30^. Colony morphology was described after observation of the strain grown after four days at 37 °C. Gram staining and spore formation were investigated and mobility was examined by microscopic observation^32^. Growth on Columbia agar at different temperatures (21 °C, 28 °C, 37 °C, 42 °C and 45 °C) and in microaerophilic conditions was tested using CampyGenTM (BioMérieux, ThermoFisher scientific) after 72 h of incubation. Growth in various NaCl concentrations (0, 5, 10 and 15 g/L) and at a pH range of 5 to 8.5 (at intervals of 0.5 pH unit) were assessed using Columbia agar plates^33^.

The ability to use mucin as sole carbon source was tested by using a modified basal media described by Derrien et al*.*^2^. Briefly, this modified medium contained 0.11 g CaCl_2_; 0.3 g NaCl; 0.53 g Na_2_HPO_4_; 0.4 g KH_2_PO_4_; 0.3 g NH_4_Cl; 0.1 g MgCl_2_.6H_2_O; 0.5 mg resazurin; 4 g NaHCO_3_; 0.25 g Na_2_S.7–9H_2_O and 1 ml of vitamin solution (0.10 mg Vitamin B12, 2.0 mg biotin, 5.0 mg riboflavin, 10.0 mg Pyridoxine–HCl, 5.0 mg Nicotinic acid, 5.0 mg p-Aminobenzoic acid, 2.0 mg Folic acid, 5.0 mg Lipoic acid, 5.0 mg D-Ca-pantothenate and 5.0 mg Thiamine-HClx 2H_2_O).

Catalase and oxidase activities were assessed by using a BBL™ DrySlide™ (Becton, Le Pont de Claix, France) according to the manufacturer’s instructions. Activities of other enzymes and metabolic characteristics were investigated by using the API ZYM, API 50CH, API 20A and API NE strips according to the manufacturer’s instructions (bioMérieux). Susceptibility to antibiotics was tested using the following E-test strip gradients: amoxicillin, benzylpenicillin, oxacillin, cefotaxine, ceftriaxone, amikacin, tobramicin, ciprofloxacin, clindamycin, doxycycline, rifampicin, vancomycin and trimethoprim-sulfamethoxazole. Plates with deposited strips were incubated at 37 °C for 48 h. Minimal inhibitory concentration (MIC) of each tested antibiotic was determined according to the manufacturer’s instructions^34^.

### Chemotaxonomic characteristics

Cellular fatty acid methyl ester (FAME) analysis was performed by Gas Chromatography/ Mass Spectrometry (GC/MS) as previously described^35^. Approximately 65 mg of bacterial biomass collected from several Columbia agar plates cultured under anaerobic conditions for 3 days at 37 °C were distributed into each of two sterile tubes. FAMEs were extracted and prepared as described before by Sasser^36^. GC/MS analyses were done as previously described^35^.

Short chain fatty acids (SCFA) were extracted and analyzed from three independent culture bottles (both blank and samples). Strain Marseille-P6666 was cultured in anaerobic blood culture vial enriched with 5% sterilized sheep blood (Becton–Dickinson, Pont de Claix, France) for three days. SCFAs were measured with a Clarus 500 chromatography system connected to a SQ8s mass spectrometer (Perkin Elmer) as previously described by Diop et al.^37^.

Polar lipid analysis of strain Marseille-P6666 was performed by Hydrophilic Interaction Liquid Chromatography-Mass Spectrometry (HILIC-MS). Total lipids were extracted from cultures plates according to the Bligh and Dyer protocol^38^. Fifty percent chloroform/methanol was used to reconstitute the chloroformed extracts previously dried under a nitrogen stream, corresponding approximately to 0.5 mg of lipid content per 100 µL (v:v). Lipid extracts were injected (5 µL) into a HILIC column (BEH HILIC, 2.1 × 100 mm, 1.7 µm, Waters, Guyancourt, France). Elution of lipids from the column was performed according to their polarity using a gradient of the following solvent compositions: A = 5% water/95% acetonitrile, B = 50% H_2_O/50% acetonitrile, both at 10 mM ammonium acetate pH8. The HD-MS method (Vion ESI-IMS-Q-TOF mass spectrometer, Waters) with positive and negative modes was used for lipid control as previously described^39^. The assignment of lipid classes was done according to the retention times (RT) of an injected standard (Splash Lipidomix, Avanti Polar Lipids, Alabaster, AL, USA). A comparison of the corresponding masses with the COMP DB LipidMAPS database (tolerance of 0.0005 *m/z*; all chains are activated) was also performed to confirm the lipid classes.

### Genome sequencing and assembly

Genomic DNAs (gDNAs) from all strains were extracted using an EZ1 biorobot and the EZ1 DNA Tissue kit (Qiagen, Hilden, Germany). The gDNAs were quantified by a Qubit assay with the high sensitivity kit (Thermofisher Scientific) to 0.2 ng/μl. gDNAs were sequenced using a MiSeq sequencer (Illumina, San Diego CA, USA) with the paired-end strategy. SPAdes was used to assemble the total reads of all genomes. Scaffolds smaller than 800 bp and those with depth values lower than 25% of the average depth (considered as possible contaminants) were deleted.

### Genome annotation and comparison

Genome annotation was performed using the Prokka software^40^. Bacterial protein-coding sequences were predicted using BLASTP (E-value of 1e-03, coverage 0.7 and identity 30%) against the Clusters of Orthologous Groups (COG) database. Graphical circular maps of genomes was generated using CGView (Circular Genome Viewer) software^41^.

As of February 22, 2022, 191 complete *A.* *muciniphila* genomic sequences were available in the NCBI GenBank database and were downloaded. For genomic comparison, we eliminated duplicate sequences, retaining only 96 complete sequences from several studies (Supplementary Table 3). Overall, a total of 114 sequences were analyzed, including eight from this study, three from other *Akkermansia* species (*A.* *glycaniphila*, "*Candidatus* A. intestinigallinarum" and "*Candidatus* A. intestinavium"), and seven from closely related species from the *Verrucomicrobiaceae* family. Several genomic comparison approaches were used to delineate the species within the *Akkermansia* genus. The Genome-to Genome Distance Calculator (https://ggdc.dsmz.de/) and PyANI (a Python package and script that provides support for calculating ANI)^42^ were used to calculate the digital DNA-DNA hybridization (dDDH) and average nucleotide identity (ANI) between studied strains, retrospectively. The pan-genome of *Akkermansia* strains was analyzed using the Roary software^43^.

### Nucleotide sequence accession numbers

The 16SrRNA gene/genome sequences are available in GenBank under accession numbers ON014381/JAMGSI000000000.1 (*Akkermansia* *massiliensis* strain Marseille-P6666), ON014382/JAMYIA000000000 (*Akkermansia* *massiliensis* strain Marseille-P9185), ON014383/JAMZOC000000000 (*Akkermansia* *muciniphila* strain Marseille-P5162), ON014384/JAMGSH000000000 (*Akkermansia* *muciniphila* strain Marseille-P7245), ON014385/JAMZOB000000000 (*Akkermansia* *muciniphila* strain Marseille-P6566), ON014386/JAMGSG000000000 (*Akkermansia* *muciniphila* strain Marseille-P9184), ON014387/JAMGSF000000000 (*Akkermansia* *muciniphila* strain Marseille-P9642) and ON014388/JAMGSE000000000 (*Akkermansia* *muciniphila* strain Marseille-Q2586). Genome sequence accession number of *Candidatus* Akkermansia timonensis AKK7 is 20100303_Bin_52_1.

### Ethical statement

Oral informed consent was obtained from all patients. The study design was validated by the ethics committees of the IHU Méditerranée Infection under number 2016-011*.*

## Supplementary Information

Supplementary Information.

Supplementary Legends.

## Abbreviations

CSUR: Collection de Souches de l’Unité des Rickettsies
DSM: Deutsche Sammlung von Mikroorganismen
MALDI-TOF MS: Matrix-assisted laser-desorption/ionization time-of-flight mass spectrometry
FAME: Fatty acid methyl ester
ORF: Open reading frame
COGs: Clusters of orthologous groups
