# Supplementary figures and images for: Reclassification of eight Akkermansia muciniphila strains and description of Akkermansia massiliensis sp. nov. and Candidatus Akkermansia timonensis, isolated from human feces
Source: Sci Rep. 2022 Dec 16;12:21747. doi: 10.1038/s41598-022-25873-0 (PMC9758162; doi:10.1038/s41598-022-25873-0)

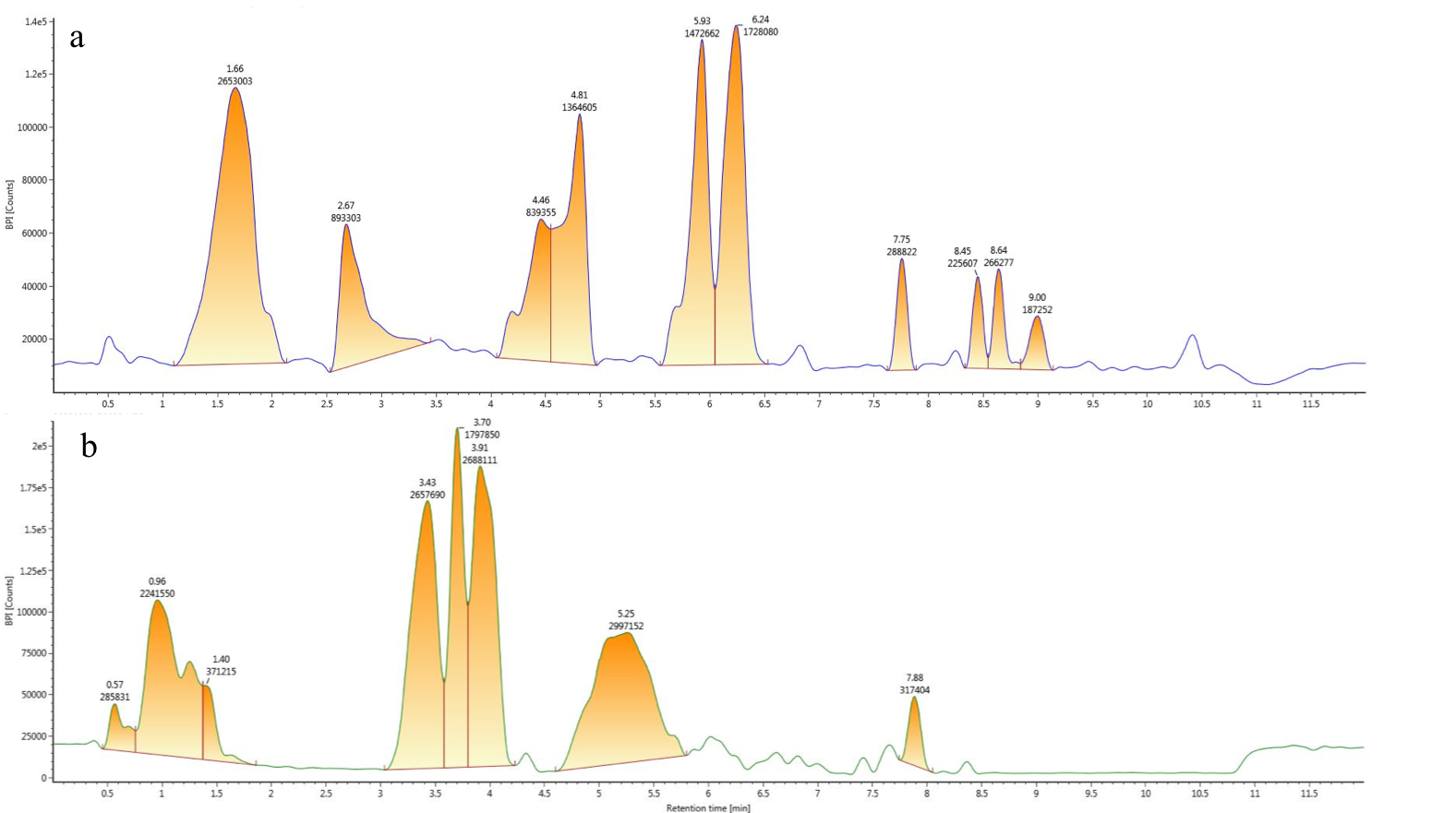

Supplement: Supplementary file 1 — Supplementary Information. [file 41598_2022_25873_MOESM1_ESM.zip › Supplementary file/Supplementary Figure 1. Polar lipids analysis of A. massiliensis Marseille-P6666T.png]
