# Supplementary material for: Reclassification of eight Akkermansia muciniphila strains and description of Akkermansia massiliensis sp. nov. and Candidatus Akkermansia timonensis, isolated from human feces
Source: Sci Rep. 2022 Dec 16;12:21747. doi: 10.1038/s41598-022-25873-0 (PMC9758162; doi:10.1038/s41598-022-25873-0)
Supplement: Supplementary file 2 — Supplementary Legends. [file 41598_2022_25873_MOESM2_ESM.docx]

**Supplementary Figure 1.** Polar lipids analysis by Hydrophilic Interaction Liquid Chromatography-Mass Spectrometry (HILIC-MS) of *Akkermansia massiliensis* strain Marseille-P6666^T^, (a): positive ionization, (b): negative ionization.

**Supplementary Figure 2**. Heatmap generated with ANI values calculated using the PyANI software between and other closely-related species.

**Supplementary Figure 3.** Phylogenetic tree based on 16S rRNA gene sequences showing the position of *Akkermansia massiliensis* strain Marseille-P6666^T^*, Akkermansia muciniphila* strain ATCC BAA-835^T^, *Akkermansia glycaniphila* strain Pyt^T^ and “*Candidatus* Akkermansia timonensis” strain Akk0196 relative to other type strains within the *Akkermansia* genus and to other members of the *Verrucomicrobiaceae* family*.* Sequences were aligned using MAFFT, and phylogenetic inferences obtained using the maximum-likelihood method within the MEGAX software. Numbers at the nodes are bootstrap values obtained by repeating the analysis 1,000 times to generate a majority consensus tree.
